# Incidence Rates of Medically Certified Long-term Sickness Absence Among Japanese Employees: A Focus on Sex Differences

**DOI:** 10.2188/jea.JE20240485

**Published:** 2025-10-05

**Authors:** Yukari Taniyama, Shohei Yamamoto, Yosuke Inoue, Toru Honda, Shuichiro Yamamoto, Tohru Nakagawa, Hiroko Okazaki, Hiroshi Ide, Toshiaki Miyamoto, Takeshi Kochi, Takayuki Ogasawara, Makoto Yamamoto, Naoki Gommori, Kenya Yamamoto, Toshitaka Yokoya, Maki Konishi, Seitaro Dohi, Isamu Kabe, Tetsuya Mizoue

**Affiliations:** 1 Department of Epidemiology and Prevention, Center for Clinical Sciences, Japan Institute for Health Security, Tokyo, Japan; 2 Hitachi Health Care Center, Hitachi Ltd, Ibaraki, Japan; 3 Mitsui Chemicals, Inc, Tokyo, Japan; 4 Nippon Steel Corporation, East Nippon Works, Chiba, Japan; 5 Furukawa Electric Co., Ltd, Tokyo, Japan; 6 Mitsubishi Fuso Truck and Bus Corporation, Kanagawa, Japan; 7 YAMAHA CORPORATION, Shizuoka, Japan; 8 East Japan Works (Keihin), JFE Steel Corporation, Kanagawa, Japan; 9 National Institute of Occupational Safety and Health Japan, Kawasaki, Kanagawa, Japan; 10 Mitsubishi Heavy Industries, Ltd, Tokyo, Japan; 11 KUBOTA Corporation, Tokyo, Japan

**Keywords:** absenteeism, incidence, occupational health, sex characteristics, sick leave

## Abstract

**Background:**

Long-term sickness absence (LTSA) is an important public health challenge, yet limited data exist on its incidence in Japan. We aimed to describe the incidence of all-cause and cause-specific LTSA by sex and age using 10-year data from a large Japanese working population, with a focus on sex differences.

**Methods:**

The study participants were employees from 16 worksites in the Japan Epidemiology Collaboration on Occupational Health Study between April 2012 and March 2022. LTSA, defined as sickness absence from work lasting 30 days or more, was recorded at each worksite. The causes of LTSA were classified using the International Classification of Diseases, 10th Revision. Incidence rates for all-cause and cause-specific LTSA were calculated based on sex and age.

**Results:**

During 730,391 and 161,513 person-years of follow-up, 6,518 and 1,866 spells of LTSA were recorded in males and females, respectively. Females had higher incidence rates of all-cause LTSA than males (115.5 vs 89.2 per 10,000 person-years), especially among females in their 20s and 30s. This was partly attributed to younger females experiencing higher LTSA incidence rates due to mental disorders, neoplasms, and pregnancy-related illnesses. In older age, females had higher LTSA incidence rates than males for musculoskeletal diseases and injuries/external causes, whereas LTSA incidence rates due to circulatory diseases were lower than those in males.

**Conclusion:**

The incidence of total and cause-specific LTSA varied greatly by sex and age, highlighting the need to consider employees’ characteristics in the prevention and management of LTSA.

## INTRODUCTION

Sickness absence (SA) reduces productivity in society, organizations, and individuals.^1^^–^^3^ The European Foundation for the Improvement of Living and Working Conditions estimated that work absences cost approximately 2.5% of GDP in Europe, totaling $470 billion in the European Union.^4^ Long-term SA (LTSA) is a significant health problem accounting for a large proportion of total SA costs. A United Kingdom survey reported that SA lasting over 10 days constituted 15% of all SA but 70% of its overall costs.^5^ LTSA also predicts adverse social and health-related risks, such as disability retirement^6^ and mortality.^7^ Descriptive data on LTSA are crucial for monitoring its incidence and guiding occupational health services for prevention and management.

The incidence of LTSA varies by employee characteristics, such as sex and age. In Europe, the incidence rates of all-cause SA (>10 days^8^ or >60 days^9^) are higher among females than males, with older individuals exhibiting higher rates, particularly among civil servants. In Japan, a study among private-sector employees found that LTSA (≥30 days) incidence rates were higher in females than males. Patterns also differed by age: in males, rates increased from the 30s, peaking in the 50s, whereas in females, the highest rates occurred in the 20s and declined with age.^10^

Data on the major causes of LTSA by sex and age can help occupational health professionals identify high-impact health problems requiring attention at different stages of working life. In Europe, mental and musculoskeletal disorders are the leading causes of LTSA.^11^ Cause-specific LTSA incidence rates by sex and age have mainly been reported for mental disorders.^12^^–^^14^ For example, a Dutch study of employees aged 20–59 found that the incidence rates of LTSA (≥28 days) due to common mental disorders were higher in females than in males across all age groups, with males aged 40–49 and females aged 30–39 showing the highest rates.^13^ Studies on Japanese employees are still limited. The National Personnel Authority reported that the leading causes of LTSA (≥30 days) among civil servants were mental disorders and neoplasms for both sexes. The third most common cause was circulatory disease among males and pregnancy-related illnesses among females, without sex- and age-specific analysis by causes.^15^ Nishiura et al reported similar patterns among private-sector employees in Japan, providing sex- and age-specific incidence rates by cause, with the analysis restricted to a 2-year period.^10^ In that study, we did not show diagnosis-specific incidence rates of LTSA and details of the sex and age differences of LTSA incidence, which could contribute to deeper insight into the disease burden at different stages of working life. Additionally, data on LTSA due to female-specific health issues remain limited. The importance of leveraging female employees has been increasingly recognized, as exemplified by the enactment of the Act on Promotion of Women’s Participation and Advancement in the Workplace in 2015.^16^ At the same time, the number of female employees in Japan has been rising, reaching 44.9% of the workforce in 2022.^17^ These trends highlight the need for real-world data on LTSA due to female-specific or female-dominant health problems.

To fill these research gaps, as an extension of Nishiura et al’s study,^10^ which analyzed LTSA during the first 2 years of the Japan Epidemiology Collaboration of Occupational Health Study (J-ECOH Study), the present study examines 10 years of data from a large Japanese working population. It investigates the incidence of all-cause and cause-specific LTSA by sex and age, focusing on sex differences.

## METHODS

### Study setting and population

The J-ECOH Study is a multicenter collaborative study examining the occurrence and determinants of common diseases among employees of various industries (eg, electric machinery and apparatus manufacturing; steel, chemical, gas, and non-ferrous metal manufacturing; automobile and instrument manufacturing; plastic product manufacturing; and healthcare). The companies participating in the J-ECOH study are mainly large enterprises with well-organized sick leave systems. They pay over two-thirds of their salary for up to 2.5 to 3.9 years while their employees use paid sick leave. Details of the J-ECOH study have been described previously.^18^^,^^19^ The study protocol was approved by the Ethics Committee of the National Center for Global Health and Medicine, Japan (no. NCGM-S-001140). While the participants did not provide verbal or written informed consent to join the study, they were allowed to refuse to participate in the study at any time. This procedure followed the Japanese Ethical Guidelines for Epidemiological Research which facilitate the procedure for obtaining consent in observational studies that use existing data.

We used data on LTSA among employees aged 20–64 years submitted by participating companies for fiscal years 2012–2021. Data included the number of employees stratified by sex and 5-year age group at each worksite during the same period. The year and period of participation varied among companies, with 16 worksites submitting data; eight participated for the longest duration of 10 years, while one participated for the shortest duration of 3 years. Electric machinery and apparatus manufacturing had the largest number of participants each year, accounting for approximately 60% of the total, ranging from 59.4% to 66.0%.

### Sickness absence registry and outcome

A study-specific SA registry was established in April 2012 to collect information on employee LTSA. Although no universal definition of LTSA exists, it is commonly defined as SA lasting at least 4 weeks.^20^ We defined LTSA as sick leave lasting 30 or more consecutive days. Occupational physicians from participating companies completed standardized forms that included employees’ sex, date of birth, LTSA causes, and start and end dates of LTSA, which were submitted to the study group. Information on LTSA causes was obtained from medical certificates written by primary physicians and submitted by employees to their workplaces when applying for paid SA. Causes were classified using the International Classification of Diseases, 10th Revision (ICD-10). The J-ECOH study group assigned the appropriate ICD-10 codes based on the Japanese Standard Disease Code Master.

The primary outcome was the incidence of LTSA between April 2012 and March 2022. Multiple LTSA spells for the same individual during the follow-up period were counted separately. We classified all LTSA spells based on the largest diagnostic categories defined by the ICD-10 chapter titles, as follows:

• Mental and behavioral disorders: F00–F99 (referred to as ‘mental disorders’).
• Injury, poisoning, and other consequences of external causes: S00–T98 (referred to as ‘injuries/external causes’).
• Physical disorders: A00–B99; C00–D48 (referred to as ‘neoplasms’); D50–D89; E00–E90; G00–G99; H00–H59; H60–H95; I00–I99 (referred to as ‘circulatory diseases’); J00–J99; K00–K93; L00–L99; M00–M99 (referred to as ‘musculoskeletal diseases’); N00–N99; O00–O99 (referred to as ‘pregnancy-related illnesses’); P00–P96; Q00–Q99; R00–R99; U00–U85; Z00–Z99.

### Statistical analysis

The incidence rate of LTSA was defined as the number of spells divided by the cumulative number of employees during the study period, with this cumulative number used as the total time at risk (person-years). Incidence rates of LTSA were expressed per 10,000 person-years and stratified by sex, age group (20–29, 30–39, 40–49, 50–59, and 60–64 years), and the largest diagnostic categories.

Trends according to sex and age group for the five most common causes of LTSA were examined. First, we aggregated the number of employees and LTSA spells by sex and age group. Second, we estimated the incidence rate ratios (IRRs) and corresponding 95% confidence intervals (CIs) using Poisson regression models. In these models, the number of LTSA spells was the dependent variable, while sex, age group, and their interaction term were the independent variables. The log of person-years was incorporated as an offset term to account for differences in the time at risk. Then, we calculated *P* values for linear trends across age groups for each sex category using orthogonal polynomial coefficients (specifically, the “contrast” command in Stata). The *P*-value for interaction between age group and sex was determined using the Wald test.

To further compare diagnoses between males and females in each of the largest diagnostic categories, the diagnosis-specific number of spells and incidence rates of LTSA were calculated by sex and age group. All statistical analyses were performed using Stata version 18.0 (Stata Corporation, College Station, TX, USA). Statistical significance was set at *P* < 0.05 for trends and *P* < 0.10 for interactions. All *P* values were two-tailed.

## RESULTS

### Incidence rates of LTSA

**Figure 1.**
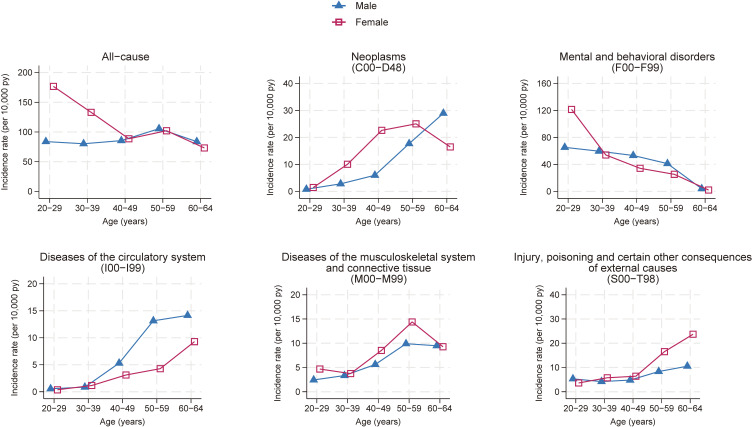
Incidence rates of long-term sickness absence for males and females across age groups by causes

As shown in Table 1, during 730,391 and 161,513 person-years of follow-up, 6,518 and 1,866 LTSA spells occurred in males and females, respectively. The overall incidence rates of all-cause LTSA were 89.2 and 115.5 spells per 10,000 person-years in males and females, respectively. Incidence rates differed by sex and age group (*P* for interaction <0.001; Table 2 and Figure 1). Females had higher incidence rates than males, with an IRR of 1.25 (95% CI, 1.17–1.34). In females, the incidence rate of LTSA was highest in the 20–29 age group (176.6 per 10,000 person-years) and decreased with age (*P* for trend <0.001). In males, the incidence rate of LTSA slightly increased from the 20–29 to 50–59 age group (*P* for trend = 0.02). Recurrence of LTSA was more common among males than females (eTable 1). In males, younger employees experienced it more frequently than older ones, while in females, there was little difference across age groups. Both males and females with LTSA due to mental disorders were more likely to experience recurrence of LTSA than those with LTSA due to other diseases.

**Table 1.** Number of spells and incidence rate of long-term sickness absence per 10,000 person-years by sex and age group from fiscal year 2012 through 2021

Largest diagnostic category	Male						Female					
	
	Number of spells (Incidence rate of LTSA [per 10,000 person-years])						Number of spells (Incidence rate of LTSA [per 10,000 person-years])					
	
	Overall	Age group, years					Overall	Age group, years				
	
		20–29	30–39	40–49	50–59	60–64		20–29	30–39	40–49	50–59	60–64
Person-years	730,391	111,281	153,060	217,162	190,905	57,983	161,513	27,809	34,791	51,693	37,517	9,703
All-cause LTSA (A00–Z99, N/A)	6,518 (89.2)	933 (83.8)	1,228 (80.2)	1,856 (85.5)	2,015 (105.5)	486 (83.8)	1,866 (115.5)	491 (176.6)	463 (133.1)	458 (88.6)	383 (102.1)	71 (73.2)
Certain infectious and parasitic diseases (A00–B99)	39 (0.5)	7 (0.6)	3 (0.2)	15 (0.7)	11 (0.6)	3 (0.5)	9 (0.6)	3 (1.1)	1 (0.3)	2 (0.4)	2 (0.5)	1 (1.0)
Neoplasms (C00–D48)	688 (9.4)	9 (0.8)	43 (2.8)	130 (6.0)	338 (17.7)	168 (29.0)	266 (16.5)	4 (1.4)	35 (10.1)	117 (22.6)	94 (25.1)	16 (16.5)
Diseases of the blood and blood-forming organs and certain disorders involving the immune mechanism (D50–D89)	10 (0.1)	0 (0.0)	2 (0.1)	4 (0.2)	4 (0.2)	0 (0.0)	7 (0.4)	0 (0.0)	4 (1.1)	1 (0.2)	2 (0.5)	0 (0.0)
Endocrine, nutritional and metabolic diseases (E00–E90)	71 (1.0)	7 (0.6)	8 (0.5)	16 (0.7)	30 (1.6)	10 (1.7)	15 (0.9)	2 (0.7)	5 (1.4)	3 (0.6)	4 (1.1)	1 (1.0)
Mental and behavioral disorders (F00–F99)	3,607 (49.4)	727 (65.3)	913 (59.6)	1,157 (53.3)	787 (41.2)	23 (4.0)	800 (49.5)	338 (121.5)	188 (54.0)	177 (34.2)	95 (25.3)	2 (2.1)
Diseases of the nervous system (G00–G99)	239 (3.3)	44 (4.0)	67 (4.4)	55 (2.5)	62 (3.2)	11 (1.9)	54 (3.3)	7 (2.5)	12 (3.4)	17 (3.3)	17 (4.5)	1 (1.0)
Diseases of the eye and adnexa (H00–H59)	39 (0.5)	4 (0.4)	3 (0.2)	8 (0.4)	19 (1.0)	5 (0.9)	12 (0.7)	0 (0.0)	1 (0.3)	1 (0.2)	8 (2.1)	2 (2.1)
Diseases of the ear and mastoid process (H60–H95)	21 (0.3)	2 (0.2)	2 (0.1)	5 (0.2)	11 (0.6)	1 (0.2)	7 (0.4)	1 (0.4)	3 (0.9)	1 (0.2)	2 (0.5)	0 (0.0)
Diseases of the circulatory system (I00–I99)	467 (6.4)	6 (0.5)	13 (0.8)	115 (5.3)	251 (13.1)	82 (14.1)	46 (2.8)	1 (0.4)	4 (1.1)	16 (3.1)	16 (4.3)	9 (9.3)
Diseases of the respiratory system (J00–J99)	64 (0.9)	9 (0.8)	16 (1.0)	6 (0.3)	25 (1.3)	8 (1.4)	11 (0.7)	2 (0.7)	0 (0.0)	5 (1.0)	4 (1.1)	0 (0.0)
Diseases of the digestive system (K00–K93)	186 (2.5)	13 (1.2)	13 (0.8)	51 (2.3)	71 (3.7)	38 (6.6)	35 (2.2)	7 (2.5)	5 (1.4)	10 (1.9)	11 (2.9)	2 (2.1)
Diseases of the skin and subcutaneous tissue (L00–L99)	27 (0.4)	3 (0.3)	5 (0.3)	9 (0.4)	6 (0.3)	4 (0.7)	6 (0.4)	2 (0.7)	1 (0.3)	0 (0.0)	1 (0.3)	2 (2.1)
Diseases of the musculoskeletal system and connective tissue (M00–M99)	444 (6.1)	27 (2.4)	51 (3.3)	122 (5.6)	189 (9.9)	55 (9.5)	133 (8.2)	13 (4.7)	13 (3.7)	44 (8.5)	54 (14.4)	9 (9.3)
Diseases of the genitourinary system (N00–N99)	58 (0.8)	5 (0.4)	8 (0.5)	20 (0.9)	18 (0.9)	7 (1.2)	25 (1.5)	3 (1.1)	6 (1.7)	8 (1.5)	6 (1.6)	2 (2.1)
Pregnancy, childbirth and the puerperium (O00–O99)	—	—	—	—	—	—	266 (16.5)	96 (34.5)	158 (45.4)	12 (2.3)	0 (0.0)	0 (0.0)
Congenital malformations, deformations and chromosomal abnormalities (Q00–Q99)	6 (0.1)	0 (0.0)	3 (0.2)	2 (0.1)	1 (0.1)	0 (0.0)	2 (0.1)	0 (0.0)	0 (0.0)	2 (0.4)	0 (0.0)	0 (0.0)
Symptoms, signs and abnormal clinical and laboratory findings, not elsewhere classified (R00–R99)	87 (1.2)	10 (0.9)	12 (0.8)	34 (1.6)	25 (1.3)	6 (1.0)	18 (1.1)	1 (0.4)	4 (1.1)	7 (1.4)	5 (1.3)	1 (1.0)
Injury, poisoning and certain other consequences of external causes (S00–T98)	447 (6.1)	59 (5.3)	64 (4.2)	104 (4.8)	159 (8.3)	61 (10.5)	148 (9.2)	10 (3.6)	20 (5.7)	33 (6.4)	62 (16.5)	23 (23.7)
Codes for special purposes (U00–U85)	4 (0.1)	1 (0.1)	0 (0.0)	0 (0.0)	1 (0.1)	2 (0.3)	1 (0.1)	0 (0.0)	0 (0.0)	1 (0.2)	0 (0.0)	0 (0.0)
Factors influencing health status and contact with health services (Z00–Z99)	14 (0.2)	0 (0.0)	2 (0.1)	3 (0.1)	7 (0.4)	2 (0.3)	4 (0.2)	1 (0.4)	2 (0.6)	1 (0.2)	0 (0.0)	0 (0.0)
N/A	—	—	—	—	—	—	1 (0.1)	0 (0.0)	1 (0.3)	0 (0.0)	0 (0.0)	0 (0.0)

LTSA, long-term sickness absence.

**Table 2.** Incident rate ratios of sex and age group by the five most common causes of long-term sickness absence from fiscal year 2012 through 2021

Largest diagnostic category	Male						Female						IRR for female to male^a^	*P* for interaction sex * age group
	
	IRR (95% CI) by age group					*P* for trend	IRR (95% CI) by age group					*P* for trend		
	
	20–29 years	30–39 years	40–49 years	50–59 years	60–64 years		20–29 years	30–39 years	40–49 years	50–59 years	60–64 years			
All-cause (A00–Z99, N/A)	Reference	0.96 (0.88–1.04)	1.02 (0.94–1.10)	1.26 (1.16–1.36)	1.00 (0.90–1.12)	0.02	Reference	0.75 (0.66–0.86)	0.50 (0.44–0.57)	0.58 (0.51–0.66)	0.41 (0.32–0.53)	<0.001	1.25 (1.17–1.34)	<0.001
Neoplasms (C00–D48)	Reference	3.47 (1.69–7.13)	7.40 (3.77–14.55)	21.89 (11.29–42.44)	35.83 (18.32–70.05)	<0.001	Reference	6.99 (2.49–19.68)	15.74 (5.81–42.63)	17.42 (6.40–47.38)	11.46 (3.83–34.29)	<0.001	1.81 (1.37–2.39)	<0.001
Mental and behavioral disorders (F00–F99)	Reference	0.91 (0.83–1.01)	0.82 (0.74–0.89)	0.63 (0.57–0.70)	0.06 (0.04–0.09)	<0.001	Reference	0.44 (0.37–0.53)	0.28 (0.23–0.34)	0.21 (0.17–0.26)	0.02 (0.00–0.07)	<0.001	0.81 (0.60–1.09)	<0.001
Diseases of the circulatory system (I00–I99)	Reference	1.58 (0.60–4.14)	9.82 (4.32–22.32)	24.39 (10.85–54.80)	26.23 (11.45–60.09)	<0.001	Reference	3.20 (0.36–28.61)	8.61 (1.14–64.90)	11.86 (1.57–89.43)	25.79 (3.27–203.60)	<0.001	0.65 (0.38–1.09)	0.15
Diseases of the musculoskeletal system and connective tissue (M00–M99)	Reference	1.37 (0.86–2.19)	2.32 (1.53–3.51)	4.08 (2.73–6.11)	3.91 (2.47–6.20)	<0.001	Reference	0.80 (0.37–1.72)	1.82 (0.98–3.38)	3.08 (1.68–5.64)	1.98 (0.85–4.64)	0.003	1.36 (1.06–1.74)	0.62
Injury, poisoning and certain other consequences of external causes (S00–T98)	Reference	0.79 (0.55–1.12)	0.90 (0.66–1.24)	1.57 (1.17–2.12)	1.98 (1.39–2.84)	<0.001	Reference	1.60 (0.75–3.42)	1.78 (0.87–3.60)	4.60 (2.36–8.96)	6.59 (3.14–13.85)	<0.001	1.41 (1.13–1.75)	0.02

CI, confidence interval; IRR, incident rate ratio.

^a^Adjusted for age group.

### LTSA due to major causes

**Figure 2.**
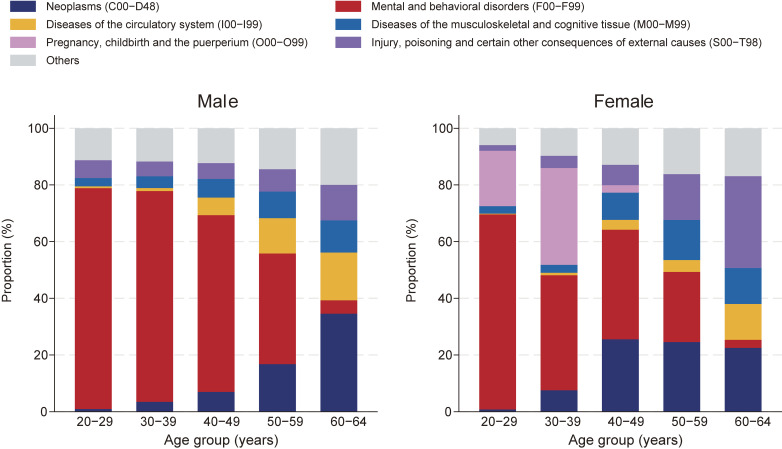
Proportion of the major causes of long-term sickness absence across age groups by sex

The five most common causes of LTSA by sex were as follows: for males, mental disorders, neoplasms, circulatory diseases, injuries/external causes, and musculoskeletal diseases; for females, mental disorders, pregnancy-related illnesses, neoplasms, injuries/external causes, and musculoskeletal diseases (Table 1). The major causes of LTSA varied across age groups (Figure 2). For both sexes, mental disorders were the leading cause among employees aged 20–59 years, although their proportion decreased with age: from 77.9% to 39.1% in males aged 20–29 and 50–59 years, respectively, and from 68.9% to 24.8% in females in the same respective age groups. Neoplasms accounted for the largest proportion of LTSA among males aged 60–64 years (34.6%), whereas injuries/external causes were the predominant cause for females in the same age group (32.4%). A notable proportion of LTSA was attributed to pregnancy-related illnesses in females aged 20–29 and 30–39 years (19.6% and 34.1%, respectively).

### Cause-specific incidence rates of LTSA: neoplasms, mental disorders, and injuries/external causes

The incidence rates of LTSA due to neoplasms, mental disorders, and injuries/external causes differed significantly by sex and age (Table 2 and Figure 1). For neoplasms, the incidence rates of LTSA were higher among females than males (IRR 1.81; 95% CI, 1.37–2.39). In males, the incidence rates increased from the 50–59 years age group (*P* for trend <0.001), whereas in females, they increased with age from the 30–39 years age group (*P* for trend <0.001). The incidence rates of LTSA due to mental disorders did not exhibit a significant difference between both sexes overall (IRR 0.81; 95% CI, 0.60–1.09), except for the 20–29 years age group, where females demonstrated higher rates. The LTSA incidence rates due to mental disorders were highest in the 20–29 years age group and declined with age for both sexes (*P* for trend <0.001 for both sexes). For injuries/external causes, the incidence rates of LTSA were higher among females than males (IRR 1.41; 95% CI, 1.13–1.75). The LTSA incidence rates due to injuries/external causes increased with age for both sexes (*P* for trend <0.001 for both sexes), particularly in females aged 50–64 years.

### Cause-specific incidence rates of LTSA: circulatory diseases and musculoskeletal diseases

The other two most common causes of LTSA, namely circulatory diseases and musculoskeletal diseases, had no significant interactions between sex and age group, but trends tended to differ by sex (Table 2 and Figure 1). For circulatory diseases, the incidence rates were lower among females than males, though the difference was not statistically significant (IRR 0.65; 95% CI, 0.38–1.09). The incidence rates of LTSA due to circulatory diseases increased with age in both sexes (*P* for trend <0.001 for both sexes). For musculoskeletal diseases, the incidence rates of LTSA were higher among females than males (IRR 1.36; 95% CI, 1.06–1.74). In both sexes, the LTSA incidence rates due to musculoskeletal diseases increased until the age of 50–59 years (*P* for trend <0.001 in males and 0.003 in females).

### LTSA due to subcategories of neoplasms

The major causes of LTSA due to neoplasms differed markedly between sexes (eTable 2). In males, colorectal (C18–C20), lung (C33–C34), and stomach (C16) cancers were the most common, accounting for 15.0%, 14.0%, and 10.8% of spells, respectively, with incidence increasing with age. In females, neoplasms of the breast and female genital organs were the predominant causes, accounting for 63.5% of LTSA spells. Breast (C50), uterine (C53–C55), and ovarian (C56) cancers contributed 23.7%, 9.4%, and 6.8%, respectively. Uterine leiomyomas (D25) and neoplasms of uncertain or unknown behavior of female genital organs (D39) were also major causes, representing 15.8% and 7.5%, respectively. Age trends revealed increasing LTSA incidence for breast, uterine, and ovarian cancers from 20 to 59 years, with uterine leiomyoma peaking in the 40–49 years age group and neoplasms of uncertain or unknown behavior of female genital organs peaking in the 30–39 years age group.

### LTSA due to subcategories of mental disorders

Approximately 80% of LTSA spells due to mental disorders for both sexes were attributable to depressive episode (F32) and reaction to severe stress and adjustment disorders (F43) (eTable 3). Depressive episode was the most common, accounting for 59.0% of spells in males and 49.1% in females. The second leading cause of LTSA for both sexes was reaction to severe stress and adjustment disorders, comprising 21.6% of spells in males and 30.3% in females. The incidence rates of LTSA due to depressive episode remained stable among males in the 20–49 years age group but declined in the 50–64 years age group. Conversely, among females, the incidence rates steadily declined with age, as did rates for reaction to severe stress and adjustment disorders for both sexes.

### LTSA due to subcategories of circulatory diseases

Cerebrovascular diseases (I60–I69) were the leading causes of LTSA due to circulatory diseases for both sexes (eTable 4). The incidence rates of LTSA due to cerebrovascular diseases were higher for males than females and began to increase after the age of 40–49 years for both sexes. Among LTSAs due to cerebrovascular diseases, cerebral infarction (I63: 24.0%) accounted for the largest proportion in males and intracerebral hemorrhage (I61: 17.4%) in females.

### LTSA due to subcategories of musculoskeletal diseases

Dorsopathies (M40–M54) were the most common causes of LTSA, with incidence rates of LTSA increasing with age from 20 to 59 years for both sexes (eTable 5). The incidence rates of LTSA due to arthropathies (M00–M25) and systemic connective tissue disorders (M30–M36) were higher among females than among males. The incidence rates of LTSA due to coxarthrosis (M16) were particularly higher among females, peaking in the 50–59 years age group.

### LTSA due to subcategories of injuries/external causes

Fractures were the most prevalent LTSA due to injuries/external causes for both sexes, accounting for 46.8% and 58.8% of spells, respectively (eTable 6). Specifically, fracture of lower leg, including ankle (S82), were the most common among males (16.8%) and females (24.3%), with incidence rates of LTSA relatively higher among females than males in the 30–64 years age group.

## DISCUSSION

The present study extended the previous work by Nishiura et al by accounting for 4.6 times greater numbers of LTSA spells in males and 6.5 times greater numbers in females. This enables us to describe sex and age differences in the incidence of all-cause and cause-specific LTSA in a large working population in Japan. Females had higher all-cause LTSA incidence rates than males, with rates declining with age in females but increasing slightly in males. Females had higher LTSA incidence rates due to neoplasms, musculoskeletal diseases, and injuries/external causes, while males had higher rates due to circulatory diseases. Notably, pregnancy-related illnesses were the second leading cause of LTSA in females.

The observed higher incidence of all-cause LTSA in females is consistent with previous studies^8^^–^^10^ and likely reflects both biological and sociological factors. Biologically, pregnancy- and reproduction-related health issues,^21^ as well as sex-specific cancers, which occur typically in the working-age population and are predominantly shaped by the hormonal influence of estrogen,^22^ contribute significantly to LTSA among females. Additionally, in females, the incidence rates of LTSA due to neoplasms began to increase in their 30s, with neoplasms of female genital organs and breast cancer accounting for a large proportion. In males, it increased markedly in their 50s, with the stomach, colorectum, and lungs as the main cancer sites, aligning with age-specific cancer incidence rates and major cancers in the working population based on 2020 data from the National Cancer Registry.^23^ These findings suggest that pregnancy-related illnesses are notable contributors to long-term labor force loss among younger females and highlight the greater impact of neoplasms on LTSA in females than in males. From a sociological perspective, some personal, family, and work-related factors might contribute to the sex differences in the incidence rates of LTSA, such as the double burden of work and caregiving,^24^ greater work-life conflicts,^25^ and inequality at work, including fewer opportunities for promotion and lower job positions,^21^ among females.

Mental disorders were the most common causes of LTSA for both sexes, with depressive episodes and reaction to severe stress and adjustment disorders being common in the present study. Females had higher LTSA incidence rates than males in the 20–29 years age group, while males had higher rates in the 30–64 years age group, and the rates decreased with age for both sexes. The higher rates in females in the 20–29 years age group align with prior studies in the Netherlands and Japan.^10^^,^^13^ However, our finding that LTSA incidence rates due to mental disorders decreased with age for both sexes contrasts with a Dutch study.^13^ This inconsistency may be attributed to the survival effect, where individuals with mental disorders tend to leave the workforce or retire earlier. Additionally, our findings differed from the 2020 Patient Survey by the Japan Ministry of Health, Labour, and Welfare, which reported the number of outpatients and inpatients with mental disorders using prevalence rather than incidence rate.^26^

We found that females had higher incidence rates of LTSA due to musculoskeletal diseases than males, particularly for osteoarthritis, reflecting previous research indicating a higher prevalence of radiographic hip osteoarthritis in females.^27^ We also found that the incidence rates of LTSA due to injuries/external causes were higher in females than in males, increasing remarkably in their 50s, mainly due to fractures. This may be partly attributed to the rapid decline in estrogen levels after menopause, which leads to osteopenia and osteoporosis,^28^ thereby increasing the risk of fractures.^29^ Our results suggest that musculoskeletal diseases and injuries/external causes impact workforce loss, especially among females aged 50 years and older.

The incidence rates of LTSA due to circulatory diseases were much lower in females than in males, consistent with community-based data on cardiovascular disease incidence in Japan.^30^ This lower incidence, particularly in females under 50 years, may partly be attributed to the lower prevalence of smoking and obesity in females^31^ and the effects of estrogen on the vasculature, which promotes vasodilation and inhibits the onset and progression of atherosclerosis.^32^ Additionally, the observed increase in the incidence of LTSA due to circulatory diseases in females over the age of 50 years, the average menopausal age in Japan,^33^ may be attributed to an increase in the risk of circulatory diseases associated with declining estrogen levels during the menopausal transition^34^ and after menopause.^35^^,^^36^

Our findings emphasize the importance of organizing occupational health services considering the sex differences in preventing and managing LTSA. Mental health care would be most crucial for younger females, showing the highest incidence of LTSA due to mental disorders. Workplace stress check programs,^37^ followed by early intervention for those with mental health problems, could help prevent LTSA due to mental disorders. Some mental health problems in females are associated with reproductive events.^38^ Thus, special care should be provided for high-risk female employees (for example, with experience of LTSA due to miscarriage^38^^,^^39^) to prevent taking another LTSA due to mental disorders. According to the priority policy on women’s participation and gender equality by the Gender Equality Bureau, Cabinet Office of Japan,^40^ questions on female-specific health issues, such as menstrual-related symptoms and menopausal disorders, will be incorporated into employer health checkups under the Industrial Safety and Health Act. Monitoring the health of female employees using such information can help occupational health professionals identify those with female-specific health problems in the early stages and thus prevent LTSA. To prevent LTSA due to musculoskeletal diseases and injuries/external causes, improving safety measures to prevent accidents at work, including the identification of high fall-risk areas and the installation of fall hazard warning signs, is a priority. Furthermore, informing female employees aged 40 years and older about community screening programs for osteoporosis^41^ would facilitate early detection, prevention, and treatment of osteoporosis, which is likely to contribute to the prevention of LTSA due to fractures.

The major strength of this study is that the source of LTSA data was derived from company SA records and medical certificates written by physicians, avoiding recall bias. This extended study also included five times more LTSA spells and a longer study period than our previous report,^10^ enabling detailed analysis, especially of female-specific diseases. However, our results should be interpreted with caution because of several limitations. First, the stigma associated with certain diagnoses may have led physicians to record alternative conditions on sick leave applications. Second, due to the lack of information on employees’ backgrounds other than sex and age, such as occupation, job positions, and physical and mental workloads, we are unable to examine whether the differences in the incidence rates of LTSA by sex and age are ascribed to these factors. Third, we started collecting data on the reasons (occupational or others) for LTSA due to external causes since 2021 and were unable to examine LTSA due to occupational accidents specifically. Fourth, the incidence rates of LTSA were higher among employees aged 50–59 years than among those aged 60–64 years, potentially reflecting the “healthy worker effect”.^42^ This effect becomes more pronounced after reaching retirement age, where healthier individuals are more likely to remain employed or be re-employed. Fifth, incidence rates of LTSA due to a specific disease do not necessarily coincide with the actual incidence or mortality rates of those diseases, and there may be a certain degree of discrepancy among them. Finally, the participating companies were invited via an occupational physician network, and they are mainly large companies with well-organized sick leave systems in secondary industries. In small- or medium-sized companies without such robust systems, the incidence rates of LTSA would be lower than those in the present study because of the greater likelihood of early retirement in the case of serious illnesses. Additionally, the findings of this study cannot be generalized to other countries, which differ in the culture of work,^43^ including the gender gap,^44^ the policy of SA,^45^ and disease susceptibility linked to racial characteristics.^46^

### Conclusions

In a large working population of Japan, females had higher LTSA incidence rates than males throughout their working lives, with notable sex- and age-related variations in high-impact health issues. Additionally, females showed greater age-related changes in LTSA incidence, reflecting distinct health challenges at different stages of their careers. Our findings highlight the importance of workplace health and safety management measures based on sex and age differences. Such measures can guide occupational health professionals, human resource managers, and policymakers in developing strategies for preventing and managing LTSA effectively. Further research is needed to examine temporal changes in LTSA incidence in relation to the coronavirus disease 2019 pandemic and to clarify background factors associated with LTSA to develop effective prevention strategies.

## SUPPLEMENTARY MATERIAL

eTable 1. Number of employees who experienced long-term sickness absence from fiscal year 2012 through 2021 by sex, age group, the five most common causes, and the number of spells

eTable 2. Number of spells and incidence rate of long-term sickness absence per 10,000 person-years due to major diagnosis among neoplasms (C00–D48) by sex and age group from fiscal year 2012 through 2021

eTable 3. Number of spells and incidence rate of long-term sickness absence per 10,000 person-years due to major diagnosis among mental and behavioural disorders (F00–F99) by sex and age group from fiscal year 2012 through 2021

eTable 4. Number of spells and incidence rate of long-term sickness absence per 10,000 person-years due to major diagnosis among diseases of the circulatory system (I00–I99) by sex and age group from fiscal year 2012 through 2021

eTable 5. Number of spells and incidence rate of long-term sickness absence per 10,000 person-years due to major diagnosis among diseases of the musculoskeletal system and connective tissue (M00–M99) by sex and age group from fiscal year 2012 through 2021

eTable 6. Number of spells and incidence rate of long-term sickness absence per 10,000 person-years due to major diagnosis among injury, poisoning and certain other consequences of external causes (S00–T98) by sex and age group from fiscal year 2012 through 2021
