# Supplementary material for: Incidence Rates of Medically Certified Long-term Sickness Absence Among Japanese Employees: A Focus on Sex Differences
Source: J Epidemiol. 2025 Oct 5;35(10):442–50. doi: 10.2188/jea.JE20240485 (PMC12420950; doi:10.2188/jea.JE20240485)
Supplement: Supplementary file 1 [file je-35-442-s001.pdf]

eTable 1. Number of employees who experienced long-term sickness absence from fiscal year 2012 through 2021 by sex, age group, the five most common causes, and the number of spells

	Number of spells	Male			Female		
		Overall	Age group, years		Overall	Age group, years	
			20–39	40–64		20–39	40–64
All-cause LTSA (A00-Z99, N/A)							
	1	4,595	1,448	3,147	1,500	782	718
	2	586	223	363	135	68	67
	3 or more	210	97	113	28	13	15
Neoplasms (C00-D48)							
	1	538	44	494	215	39	176
	2	60	5	55	17	0	17
	3 or more	10	0	10	5	0	5
Mental and behavioral disorders (F00-F99)							
	1	2,173	1,024	1,149	609	413	196
	2	392	188	204	70	41	29
	3 or more	179	86	93	15	11	4
Diseases of the circulatory system (I00-I99)							
	1	411	18	393	42	3	39
	2	25	1	24	2	1	1
	3 or more	2	0	2	0	0	0
Diseases of the musculoskeletal system and connective tissue (M00-M99)							
	1	365	64	301	95	21	74
	2	32	5	27	11	3	8
	3 or more	5	3	2	4	0	4
Injury, poisoning and certain other consequences of external causes (S00-T98)							
	1	419	116	303	133	28	105
	2	14	4	10	6	1	5
	3 or more	0	0	0	1	0	1

LTSA, long-term sickness absence.

eTable 2. Number of spells and incidence rate of long-term sickness absence per 10,000 person-years due to major diagnosis among neoplasms (C00-D48) by sex and age group from fiscal year 2012 through 2021

Diagnostic category	Diagnosis	Male						Female					
		Overall	Age group, years					Overall	Age group, years				
			20–29	30–39	40–49	50–59	60–64		20–29	30–39	40–49	50–59	60–64
Malignant neoplasms (C00-C97)		610 (8.4)	6 (0.5)	28 (1.8)	109 (5.0)	313 (16.4)	154 (26.6)	167 (10.3)	3 (1.1)	13 (3.7)	68 (13.2)	72 (19.2)	11 (11.3)
	Esophagus (C15)	28 (0.4)	0 (0.0)	0 (0.0)	4 (0.2)	17 (0.9)	7 (1.2)	1 (0.1)	0 (0.0)	0 (0.0)	0 (0.0)	1 (0.3)	0 (0.0)
	Stomach (C16)	74 (1.0)	0 (0.0)	4 (0.3)	11 (0.5)	39 (2.0)	20 (3.4)	6 (0.4)	0 (0.0)	1 (0.3)	2 (0.4)	2 (0.5)	1 (1.0)
	Colorectum (C18-C20)	103 (1.4)	1 (0.1)	5 (0.3)	23 (1.1)	51 (2.7)	23 (4.0)	18 (1.1)	0 (0.0)	1 (0.3)	5 (1.0)	7 (1.9)	5 (5.2)
	Liver and intrahepatic bile ducts (C22)	25 (0.3)	0 (0.0)	1 (0.1)	2 (0.1)	12 (0.6)	10 (1.7)	1 (0.1)	0 (0.0)	0 (0.0)	0 (0.0)	0 (0.0)	1 (1.0)
	Pancreas (C25)	37 (0.5)	0 (0.0)	2 (0.1)	7 (0.3)	22 (1.2)	6 (1.0)	3 (0.2)	0 (0.0)	0 (0.0)	1 (0.2)	1 (0.3)	1 (1.0)
	Lung (C33-C34)	96 (1.3)	1 (0.1)	5 (0.3)	9 (0.4)	56 (2.9)	25 (4.3)	11 (0.7)	0 (0.0)	0 (0.0)	3 (0.6)	8 (2.1)	0 (0.0)
	Breast (C50)	1 (0.0)	0 (0.0)	0 (0.0)	0 (0.0)	1 (0.1)	0 (0.0)	63 (3.9)	0 (0.0)	5 (1.4)	29 (5.6)	27 (7.2)	2 (2.1)
	Uterus (C53-C55)	-	-	-	-	-	-	25 (1.5)	1 (0.4)	3 (0.9)	12 (2.3)	9 (2.4)	0 (0.0)
	Ovary (C56)	-	-	-	-	-	-	18 (1.1)	0 (0.0)	1 (0.3)	10 (1.9)	7 (1.9)	0 (0.0)
	Prostate (C61)	32 (0.4)	0 (0.0)	0 (0.0)	2 (0.1)	17 (0.9)	13 (2.2)	-	-	-	-	-	-
	Other and unspecified types of non-Hodgkin lymphoma (C85)	29 (0.4)	0 (0.0)	3 (0.2)	7 (0.3)	12 (0.6)	7 (1.2)	3 (0.2)	0 (0.0)	0 (0.0)	3 (0.6)	0 (0.0)	0 (0.0)
	Myeloid leukemia (C92)	20 (0.3)	2 (0.2)	3 (0.2)	7 (0.3)	7 (0.4)	1 (0.2)	5 (0.3)	1 (0.4)	0 (0.0)	2 (0.4)	1 (0.3)	1 (1.0)
	Other malignant tumor (other than above in C00-C97)	165 (2.3)	2 (0.2)	5 (0.3)	37 (1.7)	79 (4.1)	42 (7.2)	13 (0.8)	1 (0.4)	2 (0.6)	1 (0.2)	9 (2.4)	0 (0.0)
Other neoplasms (D00-D48)		78 (1.1)	3 (0.3)	15 (1.0)	21 (1.0)	25 (1.3)	14 (2.4)	98 (6.1)	1 (0.4)	22 (6.3)	48 (9.3)	22 (5.9)	5 (5.2)
	Carcinoma in situ of cervix uteri (D06)	-	-	-	-	-	-	1 (0.1)	0 (0.0)	0 (0.0)	1 (0.2)	0 (0.0)	0 (0.0)
	Leiomyoma of uterus (D25)	-	-	-	-	-	-	42 (2.6)	0 (0.0)	12 (3.4)	23 (4.4)	7 (1.9)	0 (0.0)
	Neoplasm of uncertain or unknown behavior of female genital organs (D39)	-	-	-	-	-	-	20 (1.2)	0 (0.0)	8 (2.3)	9 (1.7)	1 (0.3)	2 (2.1)
	Neoplasm of uncertain or unknown behavior of brain and central nervous system (D43)	18 (0.2)	0 (0.0)	4 (0.3)	8 (0.4)	4 (0.2)	2 (0.3)	8 (0.5)	0 (0.0)	0 (0.0)	3 (0.6)	4 (1.1)	1 (1.0)
	Other neoplasms which are not malignant (other than above in D00-D48)	60 (0.8)	3 (0.3)	11 (0.7)	13 (0.6)	21 (1.1)	12 (2.1)	28 (1.7)	1 (0.4)	2 (0.6)	13 (2.5)	10 (2.7)	2 (2.1)

The number of new spells (Incidence rate of long-term sickness absence [per 10,000 person-years]) is presented for each diagnosis by sex and age group.

eTable 3. Number of spells and incidence rate of long-term sickness absence per 10,000 person-years due to major diagnosis among mental and behavioral disorders (F00-F99) by sex and age group from fiscal year 2012 through 2021

Diagnostic category	Diagnosis	Male						Female					
		Overall	Age group, years					Overall	Age group, years				
			20–29	30–39	40–49	50–59	60–64		20–29	30–39	40–49	50–59	60–64
Schizophrenia, schizotypal and delusional disorders (F20-F29)		74 (1.0)	7 (0.6)	20 (1.3)	30 (1.4)	17 (0.9)	0 (0.0)	14 (0.9)	1 (0.4)	6 (1.7)	5 (1.0)	2 (0.5)	0 (0.0)
	Schizophrenia (F20)	55 (0.8)	3 (0.3)	15 (1.0)	22 (1.0)	15 (0.8)	0 (0.0)	12 (0.7)	1 (0.4)	6 (1.7)	5 (1.0)	0 (0.0)	0 (0.0)
Mood [affective] disorders (F30-39)		2,368 (32.4)	414 (37.2)	589 (38.5)	768 (35.4)	581 (30.4)	16 (2.8)	415 (25.7)	167 (60.1)	101 (29.0)	90 (17.4)	56 (14.9)	1 (1.0)
	Bipolar affective disorder (F31)	140 (1.9)	12 (1.1)	23 (1.5)	65 (3.0)	38 (2.0)	2 (0.3)	13 (0.8)	5 (1.8)	1 (0.3)	5 (1.0)	2 (0.5)	0 (0.0)
	Depressive episode (F32)	2,127 (29.1)	390 (35.0)	540 (35.3)	674 (31.0)	510 (26.7)	13 (2.2)	393 (24.3)	159 (57.2)	98 (28.2)	82 (15.9)	53 (14.1)	1 (1.0)
Neurotic, stress-related and somatoform disorders (F40-48)		1,068 (14.6)	291 (26.2)	287 (18.8)	321 (14.8)	163 (8.5)	6 (1.0)	356 (22.0)	162 (58.3)	78 (22.4)	79 (15.3)	36 (9.6)	1 (1.0)
	Anxiety disorders (F40-F41)	151 (2.1)	36 (3.2)	46 (3.0)	40 (1.8)	26 (1.4)	3 (0.5)	64 (4.0)	21 (7.6)	12 (3.4)	23 (4.4)	8 (2.1)	0 (0.0)
	Reaction to severe stress, and adjustment disorders (F43)	778 (10.7)	219 (19.7)	206 (13.5)	238 (11.0)	112 (5.9)	3 (0.5)	242 (15.0)	123 (44.2)	52 (14.9)	43 (8.3)	23 (6.1)	1 (1.0)
	Somatoform disorders (F45)	68 (0.9)	15 (1.3)	20 (1.3)	18 (0.8)	15 (0.8)	0 (0.0)	16 (1.0)	6 (2.2)	3 (0.9)	5 (1.0)	2 (0.5)	0 (0.0)
Other mental and behavioral disorders (other than above in F00-F99)		97 (1.3)	15 (1.3)	17 (1.1)	38 (1.7)	26 (1.4)	1 (0.2)	15 (0.9)	8 (2.9)	3 (0.9)	3 (0.6)	1 (0.3)	0 (0.0)

The number of new spells (Incidence rate of long-term sickness absence [per 10,000 person-years]) is presented for each diagnosis by sex and age group.

eTable 4. Number of spells and incidence rate of long-term sickness absence per 10,000 person-years due to major diagnosis among diseases of the circulatory system (I00-I99) by sex and age group from fiscal year 2012 through 2021

Diagnostic category	Diagnosis	Male						Female					
		Overall	Age group, years					Overall	Age group, years				
			20–29	30–39	40–49	50–59	60–64		20–29	30–39	40–49	50–59	60–64
Heart diseases (I01-02.0, I05-I09, I20-I25, I27, I30-I52)		161 (2.2)	3 (0.3)	4 (0.3)	46 (2.1)	81 (4.2)	27 (4.7)	11 (0.7)	0 (0.0)	2 (0.6)	3 (0.6)	2 (0.5)	4 (4.1)
	Angina pectoris (I20)	20 (0.3)	0 (0.0)	0 (0.0)	5 (0.2)	14 (0.7)	1 (0.2)	1 (0.1)	0 (0.0)	0 (0.0)	0 (0.0)	1 (0.3)	0 (0.0)
	Acute myocardial infarction (I21)	38 (0.5)	0 (0.0)	1 (0.1)	10 (0.5)	19 (1.0)	8 (1.4)	2 (0.1)	0 (0.0)	0 (0.0)	1 (0.2)	1 (0.3)	0 (0.0)
	Heart failure (I50)	26 (0.4)	0 (0.0)	2 (0.1)	6 (0.3)	11 (0.6)	7 (1.2)	1 (0.1)	0 (0.0)	1 (0.3)	0 (0.0)	0 (0.0)	0 (0.0)
Cerebrovascular diseases (I60-I69)		225 (3.1)	1 (0.1)	5 (0.3)	46 (2.1)	130 (6.8)	43 (7.4)	26 (1.6)	1 (0.4)	0 (0.0)	9 (1.7)	11 (2.9)	5 (5.2)
	Subarachnoid hemorrhage (I60)	27 (0.4)	0 (0.0)	2 (0.1)	8 (0.4)	12 (0.6)	5 (0.9)	5 (0.3)	0 (0.0)	0 (0.0)	3 (0.6)	1 (0.3)	1 (1.0)
	Intracerebral hemorrhage (I61)	66 (0.9)	0 (0.0)	0 (0.0)	15 (0.7)	41 (2.1)	10 (1.7)	8 (0.5)	0 (0.0)	0 (0.0)	4 (0.8)	3 (0.8)	1 (1.0)
	Cerebral infarction (I63)	112 (1.5)	1 (0.1)	2 (0.1)	20 (0.9)	65 (3.4)	24 (4.1)	7 (0.4)	0 (0.0)	0 (0.0)	1 (0.2)	5 (1.3)	1 (1.0)
Diseases of arteries, arterioles and capillaries (I70-I79)		65 (0.9)	0 (0.0)	2 (0.1)	20 (0.9)	31 (1.6)	12 (2.1)	4 (0.2)	0 (0.0)	1 (0.3)	1 (0.2)	2 (0.5)	0 (0.0)
	Aortic aneurysm and dissection (I71)	50 (0.7)	0 (0.0)	2 (0.1)	13 (0.6)	27 (1.4)	8 (1.4)	3 (0.2)	0 (0.0)	1 (0.3)	0 (0.0)	2 (0.5)	0 (0.0)
Other diseases of the circulatory system (other than above in I00-I99)		16 (0.2)	2 (0.2)	2 (0.1)	3 (0.1)	9 (0.5)	0 (0.0)	5 (0.3)	0 (0.0)	1 (0.3)	3 (0.6)	1 (0.3)	0 (0.0)

The number of new spells (Incidence rate of long-term sickness absence [per 10,000 person-years]) is presented for each diagnosis by sex and age group.

eTable 5. Number of spells and incidence rate of long-term sickness absence per 10,000 person-years due to major diagnosis among diseases of the musculoskeletal system and connective tissue (M00-M99) by sex and age group from fiscal year 2012 through 2021

Diagnostic category	Diagnosis	Male						Female					
		Overall	Age group, years					Overall	Age group, years				
			20–29	30–39	40–49	50–59	60–64		20–29	30–39	40–49	50–59	60–64
Arthropathies (M00-M25)		78 (1.1)	2 (0.2)	6 (0.4)	18 (0.8)	40 (2.1)	12 (2.1)	48 (3.0)	2 (0.7)	3 (0.9)	9 (1.7)	28 (7.5)	6 (6.2)
	Rheumatoid arthritis (M05-M06)	17 (0.2)	1 (0.1)	2 (0.1)	3 (0.1)	10 (0.5)	1 (0.2)	10 (0.6)	0 (0.0)	1 (0.3)	1 (0.2)	7 (1.9)	1 (1.0)
	Coxarthrosis [arthrosis of hip] (M16)	13 (0.2)	0 (0.0)	0 (0.0)	4 (0.2)	8 (0.4)	1 (0.2)	23 (1.4)	0 (0.0)	2 (0.6)	2 (0.4)	17 (4.5)	2 (2.1)
	Gonarthrosis [arthrosis of knee] (M17)	24 (0.3)	0 (0.0)	0 (0.0)	3 (0.1)	15 (0.8)	6 (1.0)	10 (0.6)	0 (0.0)	0 (0.0)	5 (1.0)	3 (0.8)	2 (2.1)
Systemic connective tissue disorders (M30-M36)		21 (0.3)	4 (0.4)	3 (0.2)	8 (0.4)	4 (0.2)	2 (0.3)	15 (0.9)	5 (1.8)	2 (0.6)	5 (1.0)	3 (0.8)	0 (0.0)
	Systemic lupus erythematosus (M32)	4 (0.1)	2 (0.2)	0 (0.0)	1 (0.0)	1 (0.1)	0 (0.0)	6 (0.4)	4 (1.4)	0 (0.0)	2 (0.4)	0 (0.0)	0 (0.0)
	Dermatopolymyositis (M33)	8 (0.1)	0 (0.0)	0 (0.0)	5 (0.2)	2 (0.1)	1 (0.2)	2 (0.1)	0 (0.0)	0 (0.0)	0 (0.0)	2 (0.5)	0 (0.0)
Dorsopathies (M40-M54)		292 (4.0)	18 (1.6)	36 (2.4)	79 (3.6)	123 (6.4)	36 (6.2)	58 (3.6)	6 (2.2)	7 (2.0)	24 (4.6)	19 (5.1)	2 (2.1)
	Spondylosis (M47)	45 (0.6)	0 (0.0)	6 (0.4)	15 (0.7)	20 (1.0)	4 (0.7)	5 (0.3)	0 (0.0)	0 (0.0)	2 (0.4)	2 (0.5)	1 (1.0)
	Disc disorders (M50-M51)	146 (2.0)	14 (1.3)	23 (1.5)	46 (2.1)	50 (2.6)	13 (2.2)	28 (1.7)	4 (1.4)	4 (1.1)	16 (3.1)	4 (1.1)	0 (0.0)
	Dorsalgia (M54)	10 (0.1)	0 (0.0)	3 (0.2)	3 (0.1)	3 (0.2)	1 (0.2)	5 (0.3)	1 (0.4)	2 (0.6)	2 (0.4)	0 (0.0)	0 (0.0)
Osteopathies and chondropathies (M80-M94)		30 (0.4)	2 (0.2)	3 (0.2)	12 (0.6)	10 (0.5)	3 (0.5)	5 (0.3)	0 (0.0)	1 (0.3)	0 (0.0)	3 (0.8)	1 (1.0)
	Osteonecrosis (M87)	17 (0.2)	0 (0.0)	0 (0.0)	9 (0.4)	7 (0.4)	1 (0.2)	1 (0.1)	0 (0.0)	0 (0.0)	0 (0.0)	1 (0.3)	0 (0.0)
Other diseases of the musculoskeletal system and connective tissue (other than above in M00-M99)		23 (0.3)	1 (0.1)	3 (0.2)	5 (0.2)	12 (0.6)	2 (0.3)	7 (0.4)	0 (0.0)	0 (0.0)	6 (1.2)	1 (0.3)	0 (0.0)

The number of new spells (Incidence rate of long-term sickness absence [per 10,000 person-years]) is presented for each diagnosis by sex and age group.

eTable 6. Number of spells and incidence rate of long-term sickness absence per 10,000 person-years due to major diagnosis among injury, poisoning and certain other consequences of external causes (S00-T98) by sex and age group from fiscal year 2012 through 2021

Diagnostic category	Diagnosis	Male						Female					
		Overall	Age group, years					Overall	Age group, years				
			20–29	30–39	40–49	50–59	60–64		20–29	30–39	40–49	50–59	60–64
Dislocation, sprain and strain (S03, S13, S23, S33, S43, S53, S63, S73, S83, S93, T03, T09.2, T11.2, T13.2, T14.3)		60 (0.8)	14 (1.3)	10 (0.7)	14 (0.6)	16 (0.8)	6 (1.0)	21 (1.3)	4 (1.4)	6 (1.7)	6 (1.2)	4 (1.1)	1 (1.0)
	Joints and ligaments at neck level (S13)	18 (0.2)	1 (0.1)	2 (0.1)	6 (0.3)	7 (0.4)	2 (0.3)	7 (0.4)	0 (0.0)	4 (1.1)	2 (0.4)	1 (0.3)	0 (0.0)
	Joints and ligaments of knee (S83)	29 (0.4)	9 (0.8)	5 (0.3)	6 (0.3)	6 (0.3)	3 (0.5)	10 (0.6)	4 (1.4)	2 (0.6)	2 (0.4)	1 (0.3)	1 (1.0)
Fracture (S02, S12, S22, S32, S42, S52, S62, S72, S82, T02, T08, T10, T12, T14.2)		209 (2.9)	23 (2.1)	33 (2.2)	52 (2.4)	72 (3.8)	29 (5.0)	87 (5.4)	2 (0.7)	9 (2.6)	18 (3.5)	39 (10.4)	19 (19.6)
	Lumbar spine and pelvis (S32)	25 (0.3)	3 (0.3)	1 (0.1)	4 (0.2)	14 (0.7)	3 (0.5)	13 (0.8)	0 (0.0)	0 (0.0)	2 (0.4)	8 (2.1)	3 (3.1)
	Shoulder and upper arm (S42)	29 (0.4)	1 (0.1)	7 (0.5)	6 (0.3)	12 (0.6)	3 (0.5)	4 (0.2)	0 (0.0)	1 (0.3)	2 (0.4)	1 (0.3)	0 (0.0)
	Forearm (S52)	9 (0.1)	3 (0.3)	2 (0.1)	1 (0.0)	1 (0.1)	2 (0.3)	17 (1.1)	0 (0.0)	0 (0.0)	1 (0.2)	7 (1.9)	9 (9.3)
	Femur (S72)	22 (0.3)	2 (0.2)	1 (0.1)	8 (0.4)	5 (0.3)	6 (1.0)	9 (0.6)	1 (0.4)	0 (0.0)	1 (0.2)	6 (1.6)	1 (1.0)
	Lower leg, including ankle (S82)	75 (1.0)	6 (0.5)	18 (1.2)	17 (0.8)	25 (1.3)	9 (1.6)	36 (2.2)	1 (0.4)	8 (2.3)	9 (1.7)	14 (3.7)	4 (4.1)
	Foot, except ankle (S92)	37 (0.5)	5 (0.4)	6 (0.4)	7 (0.3)	10 (0.5)	9 (1.6)	13 (0.8)	2 (0.7)	2 (0.6)	1 (0.2)	7 (1.9)	1 (1.0)
Injury (S09.1, S16, S29.0, S39.0, S46, S56, S66, S76, S86, S96, T06.4, T09.5, T11.5, T13.5, T14.6)		46 (0.6)	1 (0.1)	5 (0.3)	14 (0.6)	21 (1.1)	5 (0.9)	11 (0.7)	1 (0.4)	2 (0.6)	2 (0.4)	6 (1.6)	0 (0.0)
	Lower leg level (S86)	28 (0.4)	1 (0.1)	5 (0.3)	10 (0.5)	10 (0.5)	2 (0.3)	6 (0.4)	0 (0.0)	1 (0.3)	1 (0.2)	4 (1.1)	0 (0.0)
Other injuries (other than above in S00-T98)		132 (1.8)	21 (1.9)	16 (1.0)	24 (1.1)	50 (2.6)	21 (3.6)	29 (1.8)	3 (1.1)	3 (0.9)	7 (1.4)	13 (3.5)	3 (3.1)

The number of new spells (Incidence rate of long-term sickness absence [per 10,000 person-years]) is presented for each diagnosis by sex and age group.
